# The two cut‐offs approach for plasma p‐tau217 in detecting Alzheimer's disease in subjective cognitive decline and mild cognitive impairment

**DOI:** 10.1002/dad2.70116

**Published:** 2025-05-11

**Authors:** Giulia Giacomucci, Chiara Crucitti, Assunta Ingannato, Valentina Moschini, Silvia Bagnoli, Federico Emanuele Pozzi, Elisa Marcantelli, Sonia Padiglioni, Carmen Morinelli, Salvatore Mazzeo, Sandro Sorbi, Valentina Berti, Benedetta Nacmias, Valentina Bessi

**Affiliations:** ^1^ Department of Neuroscience, Psychology, Drug Research and Child Health University of Florence Florence Italy; ^2^ Research and Innovation Centre for Dementia‐CRIDEM, AOU Careggi Florence Italy; ^3^ SOD Neurologia I Dipartimento Neuromuscolo‐Scheletrico e degli Organi di Senso AOU Careggi Florence Italy; ^4^ Department of Neurology, Fondazione IRCCS San Gerardo dei Tintori Monza Italy; ^5^ Milan Center for Neuroscience (NeuroMI) University of Milano‐Bicocca Milan Italy; ^6^ Regional Referral Centre for Relational Criticalities Tuscany Region Florence Italy; ^7^ Vita‐Salute San Raffaele University Milan Italy; ^8^ IRCCS Policlinico San Donato San Donato Milanese Italy; ^9^ IRCCS Fondazione Don Carlo Gnocchi Florence Italy; ^10^ Department of Biomedical Experimental and Clinical Sciences “Mario Serio,” University of Florence Florence Italy; ^11^ Nuclear Medicine Unit Azienda Ospedaliero‐Universitaria Careggi Florence Italy

**Keywords:** Alzheimer's disease, mild cognitive impairment, plasma biomarkers, plasma phosphorylated tau 217, subjective cognitive decline

## Abstract

**BACKGROUND:**

The study aimed to explore the applicability of plasma phosphorylated tau (p‐tau)217 in identifying patients with subjective cognitive decline (SCD) and mild cognitive impairment (MCI) carrying Alzheimer's disease (AD) pathology in real‐world settings.

**METHODS:**

Fifty SCD, 87 MCI, and 50 AD‐demented patients underwent blood collection to dose plasma p‐tau217 with a fully automated Lumipulse G600II assay. Patients were classified according to the Revised Criteria of the Alzheimer's Association Workgroup as Core1+ or Core1– (based on amyloid positron emission tomography, cerebrospinal fluid [CSF] amyloid beta [Aβ]42/Aβ40, CSF p‐tau181/Aβ42).

**RESULTS:**

Plasma p‐tau217 was accurate for discriminating between Core1+ and Core1– patients (area under the curve = 0.92) with an optimal cut‐off value of 0.274 pg/mL, revealing good accuracy (86.29%), positive predictive value (PPV; 88.18%), and negative predictive value (NPV; 83.09%). The two cut‐offs approach (0.229–0.516 pg/mL) showed higher accuracy (91.11%), a PPV of 96.25% and a NPV of 83.63%.

**CONCLUSION:**

The two cut‐offs approach provides for stronger accuracy, PPV, and NPV than a single cut‐off, making reliable the clinical application of plasma p‐tau217 for early detection of AD in real‐world settings.

**Highlights:**

- Plasma phosphorylated tau (p‐tau)217 was highly accurate in detecting Alzheimer's disease (AD) pathology.
- The two cut‐offs approach increased plasma p‐tau217 accuracy for AD diagnosis.
- Even when measured with immunoassay, p‐tau217 is a good biomarker for AD diagnosis.
- Transition of p‐tau217 from research setting to clinical practice seems feasible.

## 1 INTRODUCTION

Recent advances in novel and highly accurate blood‐based biomarkers (BBMs) may soon facilitate early and accessible diagnosis of Alzheimer's disease (AD) in the clinical setting.^1^ Such credit has been given to BBMs that plasma phosphorylated tau (p‐tau) measurements have been officially included in the most recent revision of the Revised Criteria of the Alzheimer's Association Workgroup for diagnosing and staging AD.^2^ This innovation, along with the recent approval of specific disease‐modifying treatments (DMTs) targeting amyloid beta (Aβ) by the European Medicines Agency (EMA)—after earlier approvals in the United States, Japan, and the UK—highlights the need to accurately identify individuals with AD pathology who are still in the early stages of the disease, to ensure they can benefit clinically from treatment.^3^

Plasma measurements of p‐tau have proven to be particularly accurate in distinguishing patients with neuropathologically confirmed AD from patients with other causes of cognitive impairment.^4^, ^5^, ^6^ Among its various isoforms, plasma p‐tau217 has garnered the most interest in the scientific community. According to the new Revised Criteria, p‐tau217 has been proposed as a T1 biomarker, representative of soluble tau fragments that may reflect a reaction to amyloid plaques or to soluble Aβ species in plaque penumbra, and so a very first stage of phosphorylation and secretion of tau induced by amyloid pathology.^2^ Indeed, there is growing evidence that plasma p‐tau217 is an early marker of AD, as it can be considered an amyloid response measure.^7^ Moreover, plasma p‐tau217 levels start to elevate early in the disease process, during the pre‐symptomatic stages, in contrast to tau positron emission tomography (PET), which changes predominantly during the later stages of the disease.^6^, ^8^, ^9^, ^10^ Furthermore, some studies suggested that plasma p‐tau217 may even be useful in the earliest phase of the disease's continuum, that is, subjective cognitive decline (SCD).^11^, ^12^.

Nevertheless, to make plasma biomarkers actually applicable outside of the research setting, it is crucial to address some issues regarding their exploitation in clinical practice. These include developing simple and widely available diagnostic methods and commercial tools,^13^, ^14^, ^15^ establishing clear cut‐off values for identifying AD pathology and predicting conversion to AD dementia in the general population,^16^ and validating these thresholds in prodromal and preclinical stages like mild cognitive impairment (MCI) and SCD, where AD pathology prevalence is lower, in both primary and secondary care.^12^ In this ever‐changing setting, our study aims: (1) to compare plasma p‐tau217 levels in real‐world memory clinic patients across SCD, MCI, and AD dementia; (2) to assess plasma p‐tau217 diagnostic accuracy in detecting AD pathology based on the Revised Criteria; (3) to establish a single diagnostic cut‐off value and evaluate its concordance with established biomarkers; (4) to explore a two cut‐offs approach to improve diagnostic precision for clinical application in memory clinics.

## 2 MATERIALS AND METHODS

### 2.1 Participants

Between July 2018 and September 2024, we consecutively enrolled 187 patients (50 SCD, 87 MCI, and 50 AD dementia) referred to the Centre for AD and Adult Cognitive Disorders of Careggi Hospital in Florence.

Patients met the following inclusion criteria:

1. A clinical diagnosis of AD dementia according to the National Institute on Aging–Alzheimer's Association (NIA‐AA) criteria, including the atypical variants.^17^
2. A clinical diagnosis of MCI according to NIA‐AA criteria.^18^
3. A clinical diagnosis of SCD according to SCD‐I criteria.^19^

Exclusion criteria were: history of head injury, current neurological and/or systemic disease, symptoms of psychosis, major depression, substance use disorder.

At baseline, patients underwent comprehensive family and clinical history, neurological examination and extensive neuropsychological investigation (described in detail elsewhere^20^), blood collection for measurement of plasma p‐tau217 concentration, and genetic analysis.

Age at onset was defined as the age at which the patient first began experiencing cognitive symptoms. A positive family history of dementia was defined as having one or more first‐degree relatives with a documented history of cognitive decline.

Renal function was categorized as either impaired or not impaired based on estimated glomerular filtration rate (eGFR; considered impaired if < 60 mL/min/1.73 m^2^). eGFR values were collected only in patients with renal failure.

Apolipoprotein E (*APOE*) genotyping was available for 174 patients (47 SCD, 82 MCI, 50 AD dementia). Patients underwent assessment of AD biomarkers: cerebrospinal fluid (CSF) biomarker analysis was performed as the first‐line approach, while amyloid PET was used in those who refused lumbar puncture. In particular, 169 patients (36 SCD, 83 MCI, 50 AD dementia) underwent CSF collection for Aβ42, Aβ42/Aβ40, total tau (t‐tau), p‐tau, and p‐tau/Aβ42. Normal values for CSF biomarkers were: Aβ42 > 670 pg/mL, Aβ42/Aβ40 > 0.062, t‐tau < 400 pg/mL, p‐tau < 60 pg/mL, p‐tau/Aβ42 < 0.068.^21^ Forty‐three patients (23 SCD, 16 MCI, and 4 AD dementia) underwent amyloid PET scans. Both CSF collection and amyloid PET scans were performed in 31 patients (15 SCD, 12 MCI, and 4 AD dementia). Methods for CSF collection and analysis, and amyloid PET acquisition are described in Materials S1 in supporting information.

### 2.2 Classification of patients according to the Revised Criteria of the Alzheimer's Association Workgroup

Based on biomarker results, patients were classified according to the Revised Criteria of the Alzheimer's Association Workgroup. Patients were rated as Core1+ in the case of abnormality on at least one of the Core1 biomarkers (amyloid PET, CSF Aβ42/Aβ40, CSF p‐tau 181/Aβ42), and as Core1– in the case of normal Core1 biomarkers.^2^ Patients were further classified according both to diagnosis (SCD, MCI, AD dementia) and Core1 biomarker results as follows: SCD Core1– (*n* = 25), SCD Core1+ (*n* = 13), MCI Core1– (*n* = 42), MCI Core1+ (*n* = 45), AD dementia (*n* = 50, all Core1+).

### 2.3 Plasma p‐tau217

Blood samples were collected by venipuncture into standard polypropylene ethylenediaminetetraacetic acid test tubes (Sarstedt). Plasma was isolated from peripheral blood samples within 2 hours of collection. Blood samples were centrifugated at 1300 g for 15 minutes. One mL plasma aliquots were pipetted into polypropylene cryotubes and stored at −80°C until further testing. Plasma p‐tau‐217 levels were measured using fully automated CLEIA on the LUMIPULSE G600II system according to the manufacturer's guidelines (Lumipulse assay, Fujirebio, RUO for research use only).

**RESEARCH IN CONTEXT**

1. **Systematic review**: The recent development of accurate and minimally invasive blood biomarkers, such as plasma phosphorylated tau (p‐tau)217, stands to facilitate early detection and treatment of Alzheimer's disease (AD). However, to make plasma p‐tau217 actually applicable outside of the research setting, it is essential to address some issues regarding its exploitation in clinical practice.
2. **Interpretation**: Our findings suggest that the use of a two cut‐offs approach improved the accuracy of plasma p‐tau217, aligning with the recommendations by the Global CEO Initiative on AD regarding the required performance of blood‐based biomarkers for diagnosing AD.
3. **Future directions**: Plasma p‐tau217, even when measured with immunoassay, is an excellent diagnostic biomarker for AD, and it should be included in the decision‐making algorithm that considers the clinical context. This article strongly supports the feasibility of plasma p‐tau217 in real‐world memory clinics and its transition from the research setting to clinical practice.

### 2.4 Statistical analysis

All statistical analyses were performed using IBM SPSS Statistics software version 25 (SPSS Inc.), Jamovi (Jamovi version 2.3), and the computing environment R4.2.3 (R Foundation for Statistical Computing). All *p* values were two tailed, and the significance level for all analyses was set at *p *= 0.05. Distributions of all variables were assessed using the Shapiro–Wilk test. We conducted descriptive statistics using means and standard deviation for continuous variables and frequencies or percentages and 95% confidence intervals (CIs) for categorical variables (*APOE* genotype, family history of AD). Differences among groups in continuous variables were assessed through one‐way analysis of variance followed by a Bonferroni post hoc test. To adjust for possible confounding factors (age), we used analyses of covariance. Non‐parametric Spearman *ρ* (rho) was used to evaluate correlations between groups’ numeric measures (plasma p‐tau217 levels, CSF biomarkers levels, age). Chi‐squared tests were run to compare categorical data. Effect sizes were computed using partial *η* ^2^, Cohen *d*, and Cramer V. A multiple regression analysis considering diagnosis (SCD, MCI, or AD dementia), age at plasma collection, Core 1 status, and *APOE* genotypes as covariates was run to assess which variables independently influenced plasma p‐tau217 levels.

Receiver operating characteristic (ROC) analyses were performed to evaluate the ability of plasma p‐tau217 to distinguish between Core1+ and Core1– patients. We used two approaches to categorize patients based on plasma p‐tau217 levels. First, we created two groups (that is, positive and negative) based on an optimal cut‐off, which was calculated considering the maximum sum of sensitivity and specificity as the metric, evaluating accuracy, and positive and negative predictive values (PPV and NPV). Cohen *k* was used to explore concordance between plasma p‐tau217 and Core 1 status. In a second approach, we created three groups of participants (that is, positive, negative, and intermediate) to classify them as Core1+ and Core1–, using two different thresholds as previously described.^22^ According to the Global CEO initiative on AD, the performance of confirmatory BBM test should have ≈ 90% sensitivity and specificity, with no more than 15% to 20% of results with intermediate values.^23^ To meet the simultaneous goal of sensitivity and specificity ≥ 90% and an intermediate results percentage < 20%, we examined three selected combinations of sensitivity and specificity that achieved this goal: both sensitivity and specificity fixed at 95% (95/95); sensitivity fixed at 92% and specificity at 96% (92/96); both sensitivity and specificity fixed at 90%^24^ (Materials S2 in supporting information).

## 3 RESULTS

### 3.1 Distribution of plasma p‐tau217 across diagnostic and Core1 groups

**FIGURE 1:**
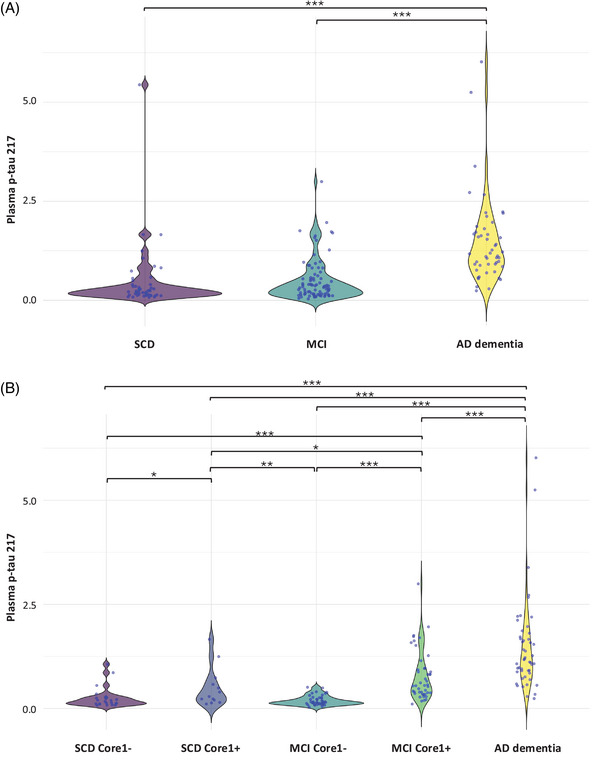
Plasma p‐tau217 levels according to clinical and clinical–biological diagnosis. A, Plasma p‐tau217 levels across diagnostic groups. Values quoted in the *y* axis indicate plasma p‐tau217 levels. Horizontal bars indicate significant differences between groups. B, Plasma p‐tau217 levels across diagnostic/Core1 groups. Values quoted in the *y* axis indicate plasma p‐tau217 levels. Horizontal bars indicate significant differences between groups. *p < 0.05; **p < 0.01; ***p < 0.001. AD, Alzheimer's disease; MCI, mild cognitive impairment; p‐tau, phosphorylated tau; SCD, subjective cognitive decline

Demographic variables are described in Table 1 and in Materials S2 (Table S1 in supporting information). Plasma p‐tau217 levels were different among the groups (*F* [4,170] = 15.87, *p *< 0.001, partial *η* ^2 ^= 0.272), also after adjusting for age (*F* [4,158] = 13.32, *p *< 0.001, partial *η* ^2 ^= 0.252). In more detail, AD dementia patients showed the highest plasma p‐tau217 concentration. MCI Core1+ had higher plasma p‐tau217 levels than MCI Core 1– (*p* < 0.001); similarly, plasma p‐tau217 levels were higher in SCD Core1+ than in SCD Core1– (*p *= 0.023; Figure 1A and 1B). To analyze which factors might influence plasma p‐tau217 levels, we ran a multiple backward regression analysis, which significantly predicted plasma p‐tau1217 levels (*F* [3,159] = 14.89, *p *< 0.001, adj. *R* ^2 ^= 0.205). Among the covariates, both diagnosis (*β* = 0.398 [95% CI: 0.146–0.651], *p  *= 0.002) and Core 1 status (*β* = 0.746 [95% CI: 0.360–1.133], *p *< 0.001) added statistical significance to the prediction. When we repeated the same analysis only in SCD and MCI groups, the model predicted plasma p‐tau1217 levels again (*F* [1,115] = 42.91, *p *< 0.001, adj. *R* ^2 ^= 0.265), but only Core1 status was significantly associated with plasma p‐tau217 levels (*β* = 0.668 [95% CI: 0.466–0.870], *p *< 0.001; Table S2 in supporting information).

**TABLE 1:** Demographic features of diagnostic and biomarkers groups.

	SCD Core1–	SCD Core1+	MCI Core1–	MCI Core1+	AD dementia
	*n* = 25	*n* = 13	*n =* 42	*n =* 45	*n =* 50
Age at onset in years	**56.52 (±7.61)** ^a^, ^b^, ^c^	**57.00 (±10.96)** ^d^, ^e^	**63.14 (±10.74)** ^a^	**67.42 (±8.49)** ^b^, ^d^	**67.57 (±6.56)** ^c^, ^e^
Age at plasma collection	**63.31 (±6.35)** ^f^, ^g^	68.41 (±8.70)	68.85 (±8.68)	**70.89(±8.42)** ^f^	**70.63 (±6.84)** ^g^
Family history of AD	80.00% (64.32–95.68)	84.60% (64.08–100)	57.90% (42.20–73.60)	62.80% (48.35–77.25)	56.80% (42.16–71.44)
Sex (M–F)	7–18	6–7	18–24	18–27	21–29
Years of education	13.79 (±3.42)	13.15 (±4.12)	11.76 (±4.68)	12.35 (±4.70)	10.83 (±4.73)
MMSE	**29.21 (±0.88)** ^h^, ^i^	28.31 (±1.25)	27.49 (±2.12)	**26.30 (±2.75)** ^h^	**19.56 (±4.90)** ^i^
*APOE* ɛ4+	**26.10%** ^j^, ^k^ **(8.15‐44.05)**	**15.40%** ^l^, ^m^ **(1.92‐45.45)**	**20.00%** ^n^, ^o^ **(7.60‐32.40)**	**59.50%** ^j^, ^l^, ^n^ **(44.65‐74.35)**	**53.30%** ^k^, ^m^, ^o^ **(38.93‐68.07)**
Impaired renal function	0	0	0	0	1 (2.00%)
Plasma p‐tau217 (pg/mL)	**0.24 (±0.24)** ^p^, ^q^, ^r^	**0.49 (±0.47)** ^p^, ^s^, ^t^, ^u^	**0.19 (±0.11)** ^q^, ^s^, ^v^, ^w^	**0.78 (±0.59)** ^t^, ^v^, ^x^	**1.65 (±1.70)** ^r^, ^u^, ^w^, ^x^

*Note*: Values are reported as mean and standard deviation or frequencies or percentages for continuous variables and categorical variables respectively. Statistically significantly different values between the groups are reported in **bold**. Statistical significance: *pp *< 0.05.

Abbreviations: AD, Alzheimer's disease; *APOE*, apolipoprotein E; F, females; M, males; MCI, mild cognitive impairment; MMSE, Mini‐Mental State Examination; p‐tau, phosphorylated tau; SCD, subjective cognitive decline.

^a^ *p *= 0.03;

^b^ *p *< 0.001;

^c^ *p *< 0.001;

^d^ *p *= 0.002;

^e^ *p *< 0.001;

^f^ *p *= 0.002;

^g^ *p *= 0.002;

^h^ *p *= 0.005;

^i^ *p *< 0.001;

^j^ *χ* ^2^ 6.66, *p *= 0.018;

^k^ *χ* ^2^ 4.58, *p* = 0.041;

^l^ *χ* ^2^ 7.74, *p *= 0.010;

^m^ *χ* ^2^ 5.87, *p *= 0.025;

^n^ *χ* ^2^ 13.31, *p *< 0.001;

^o^ *χ* ^2^ 10.03, *p *= 0.002;

^p^ *p* = 0.023;

^q^ *p* < 0.001;

^r^ *p* < 0.001;

^s^ *p* = 0.005;

^t^ *p* = 0.039;

^u^ *p* < 0.001;

^v^ *p* < 0.001;

^w^ *p* < 0.001;

^x^ *p* < 0.001.

### 3.2 Correlation between plasma p‐tau217 and CSF biomarkers

**FIGURE 2:**
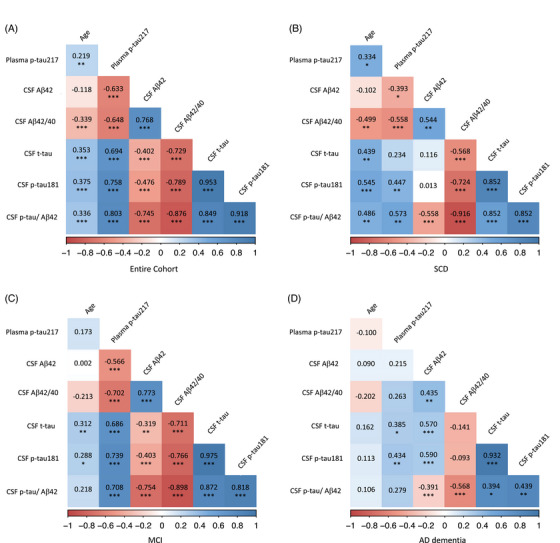
Correlation matrices. A, Correlations in entire cohort. B, Correlations in SCD subgroups. C, Correlations in MCI subgroups. D, Correlations in AD dementia subgroups. Values quoted in the correlation matrix are Spearman *ρ* correlation coefficients. Statistical significance: *P *< 0.05. Color maps represent Spearman *ρ* correlation coefficients. *p < 0.05; **p < 0.01; ***p < 0.001. Aβ, amyloid beta; AD, Alzheimer's disease; CSF, cerebrospinal fluid; MCI, mild cognitive impairment; SCD, subjective cognitive decline; p‐tau, phosphorylated tau

In the entire cohort, plasma p‐tau217 levels were significantly correlated with Aβ42 (*ρ* = −0.633, *p *< 0.001), Aβ42/1–40 ratio (*ρ* = −0.648, *p *< 0.001) and p‐tau (*ρ* = 0.758, *p *< 0.001), t‐tau (*ρ* = 0.694, *p *< 0.001) and p‐tau/Aβ42 (*ρ* = 0.803, *p *< 0.001; Figure 2A). Considering single diagnostic subgroups, in SCD patients plasma p‐tau217 levels were significantly correlated with Aβ42 (*ρ* = −0.393, *p *= 0.018), Aβ42/1–40 ratio (*ρ* = −0.558, *p *< 0.001), and p‐tau (*ρ* = 0.447, *p *= 0.006) and p‐tau/Aβ42 (*ρ* = 0.537, *p *= 0.001; Figure 2B). Similarly, in MCI patients, plasma p‐tau217 levels were significantly correlated with Aβ42 (*ρ* = −0.566, *p *< 0.001), Aβ42/1–40 ratio (*ρ* = −0.702, *p *< 0.001) and p‐tau (*ρ* = 0.739, *p *< 0.001), t‐tau (*ρ* = 0.686, *p *< 0.001), and p‐tau/Aβ42 (*ρ* = 0.780, *p *< 0.001; Figure 2C). Finally, in AD dementia plasma p‐tau217 was significantly correlated with p‐tau (*ρ* = 0.434, *p *= 0.006) and t‐tau (*ρ* = 0.385, *p *= 0.016; Figure 2D).

### 3.3 Plasma p‐tau 217 accuracy in predicting Core1 status and single cut‐off approach

**FIGURE 3:**
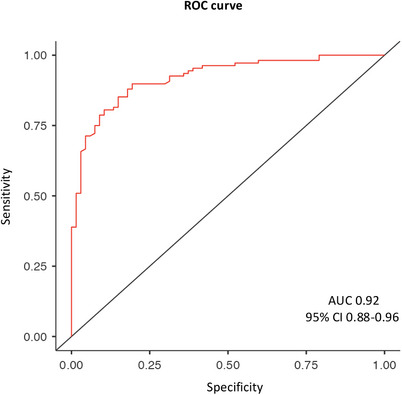
ROC curves for accuracy of plasma p‐tau217 in distinguishing Core1+ from Core1– patients. AUC, area under the curve; CI, confidence interval; ROC, receiver operating characteristic; p‐tau, phosphorylated tau

**FIGURE 4:**
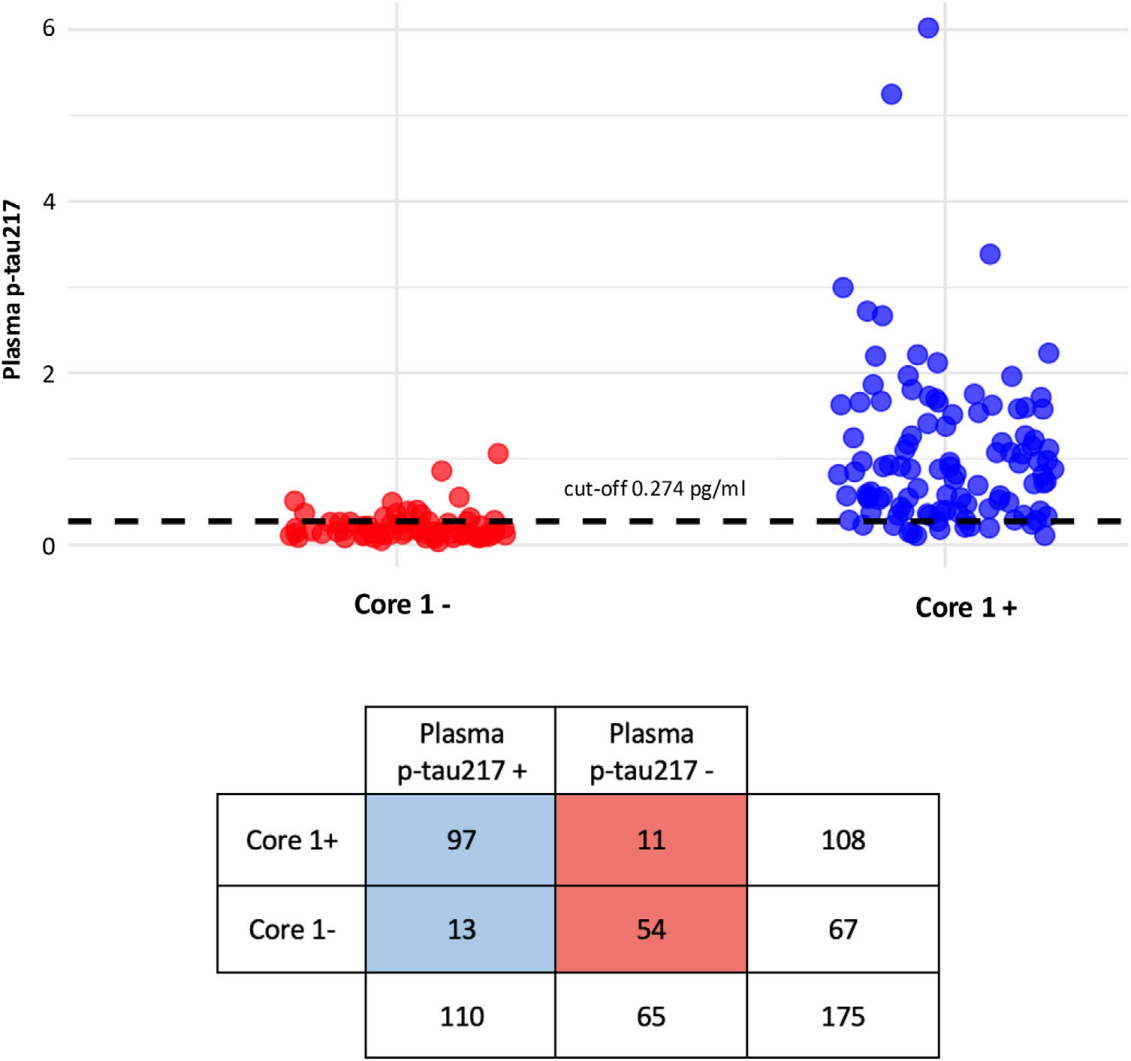
Single cut‐off approach in discriminating Core1+ from Core1– patients. Dot plot illustrating distribution of patients based of plasma p‐tau217 levels categorization according to single cut approach. The *x* axis represents Core1 status, the *y* axis represents plasma p‐tau217 levels. Blue dots: patients with plasma p‐tau217 levels above the cut‐off. Red dots: patients with plasma p‐tau217 levels below the cut‐off. p‐tau, phosphorylated tau

We performed a ROC curve analysis to evaluate the diagnostic accuracy of plasma p‐tau217 in distinguishing between Core1+ and Core1– patients. The analysis showed that plasma p‐tau217 was highly accurate for discriminating between Core1+ and Core1– patients (area under the curve [AUC] = 0.92 [95% CI: 88.00–96.00]; Figure 3). Optimal cut‐off value of 0.274 pg/mL discriminated Core1+ from Core1– patients with good accuracy (86.29% [95% CI: 81.20–91.39]), sensitivity (89.81% [95% CI: 85.33–94.3]), specificity (80.60% [95% CI: 77.44–84.46]), PPV 88.18% ([95% CI: 83.40–92.96]), and NPV (83.09% [95% CI: 77.52–88.63]; Figure 4).

According to the optimal cut‐off, plasma p‐tau217 levels were dichotomized as positive (p‐tau217 ≥ 0.274 pg/mL) and negative (p‐tau217 < 0.274 pg/mL). Cohen *K* was significant (*p *< 0.001) with a value of 0.71, indicating a good concordance between plasma p‐tau217 and Core1 status. Plasma p‐tau217 and Core1 status were concordant in 86.28% (95% CI: 81.18–91.38) of cases, with both positive plasma p‐tau217 and Core1 status in 97 cases (true positivity 55.42%) and with both negative plasma p‐tau217 and Core1 status in 54 cases out of 175 patients (true negativity 30.85%). On the other hand, plasma p‐tau217 and Core1 status were discordant in 13.71% of cases: among these, 11 out of 24 were Core1+ but showed negative plasma p‐tau217 (false negativity 6.28%), while 13 cases were Core1– but exhibited positive plasma p‐tau217 (false positivity 7.42%).

### 3.4 Two cut‐off approach

**FIGURE 5:**
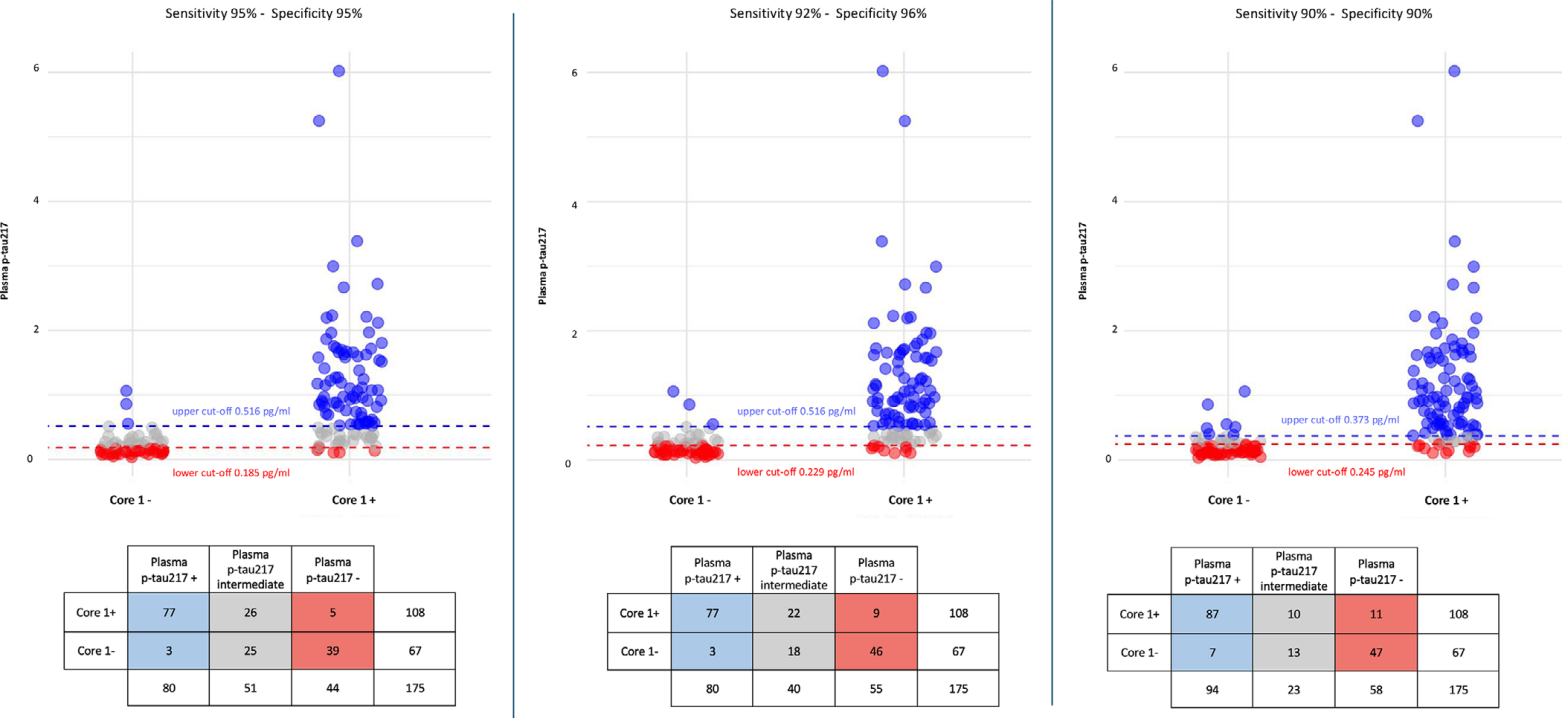
Two cut‐offs approach in discriminating Core1+ from Core1– patients. Dot plot illustrating distribution of patients based of plasma p‐tau217 levels categorization according to two cut‐offs approach using three different combinations of sensitivity and specificity: both sensitivity and specificity fixed at 95% (95/95; left); sensitivity fixed at 92% and specificity at 96% (92/96; middle); both sensitivity and specificity fixed at 90% (right). The *x* axis represents Core1 status, the *y* axis represents plasma p‐tau217 levels. Blue dots: patients with plasma p‐tau217 levels above the upper cut‐off (clearly positive). Red dots: patients with plasma p‐tau217 levels below the lower cut‐off (clearly negative). Gray dots: patients with intermediate values of plasma p‐tau217 (gray zone). p‐tau, phosphorylated tau

To evaluate potential improvements in diagnostic accuracy, we applied an approach with two cut‐offs, dividing results into three categories: those with clearly normal values, those with clearly abnormal values, and those with intermediate values (gray zone). Results are shown in Figure 5.

Considering the first combination (95/95), the upper cut‐off (specificity of 95%) was 0.516 pg/mL, and the lower cut‐off (sensitivity of 95%) was 0.185 pg/mL. Plasma p‐tau217 had an overall excellent accuracy of 93.55% (95% CI: 89.22–97.87), a PPV of 96.25% (95% CI: 92.91–99.59), and a NPV of 88.64% (95% CI: 83.05–94.22) in predicting Core1 status. The gray zone group included 29.14% of patients (15 SCD, 33 MCI, and 3 AD dementia): 50.98% of them were Core1+. Considering the second combination (92/96), the upper cut‐off (specificity of 96%) was 0.516 pg/mL, while the lower cut‐off (sensitivity of 92%) was 0.229 pg/mL. Also in this case, plasma p‐tau217 showed an overall excellent accuracy in predicting Core1 status (91.11% [95% CI: 86.31–95.91]), a PPV of 96.25% (95% CI: 93.05–99.45]), and a NPV of 83.63% (95% CI: 77.40–89.88). In this case, the grey zone included 22.85% of patients (10 SCD, 27 MCI, 3 AD dementia), of whom 55% were Core1+. Finally, with the last combination (90/90), the upper cut‐off (specificity of 90%) was 0.373 pg/mL, while the lower cut‐off (sensitivity of 90%) was 0.245 pg/mL. Plasma p‐tau217 levels discriminated Core1+ from Core1– patients with good accuracy (88.15% [95% CI: 83.02–93.29]), a PPV of 92.55% (95% CI: 88.38–96.73]), and a NPV of 81.03% (95% CI: 74.80–87.27). In this case, 13.14% of patients (6 SCD, 15 MCI, 2 AD dementia) presented intermediate plasma p‐tau217 values; 43.47% of them were Core1+.

## 4 DISCUSSION

The recent publication of the Revised Criteria of the Alzheimer's Association Workgoup focused on the use of plasma biomarkers for diagnosis of AD, thereby suggesting a migration from more invasive and expensive assays to more accessible tools.^2^ Current research agrees that plasma p‐tau217 appears to be the most promising BBM, as it has been identified as the strongest predictor of the neuropathological hallmarks of AD.^6^, ^25^ Although it has been previously demonstrated that plasma p‐tau217 is undoubtfully accurate in detecting AD pathology even in the earliest stage of the disease,^6^, ^11^, ^12^, ^26^, ^27^ fewer studies have evaluated p‐tau217 in real‐world memory clinic settings,^16^, ^28^ one of the most important contexts for the BBM application in AD diagnosis and in determining eligibility for anti‐amyloid DMTs.

Our study perfectly aligns with this perspective, demonstrating that, thanks to the use of a two cut‐offs approach, plasma p‐tau217 is an excellent biomarker for detecting AD pathology—defined according to the Revised Criteria—in a real‐world setting, in the earliest phases of cognitive decline, such as SCD and MCI.

Considering SCD and MCI, plasma p‐tau217 levels were higher among those with positive Core1 biomarkers, thus with an underlying AD pathology. This is consistent with previous findings recently reporting higher plasma p‐tau217 levels in Aβ carriers than in non‐carriers.^6^, ^29^ Moreover, patients with AD dementia exhibited the highest plasma p‐tau217 concentrations, in line with recent literature showing that plasma p‐tau217 levels increase over time in Aβ‐positive patients, reaching their peak during the dementia stage.^27^ As expected, the regression analysis revealed that plasma p‐tau217 was significantly influenced by Core1 biomarker positivity, further supporting the strong dependence of this plasma biomarker to the underlying AD pathology.^30^

Furthermore, in our real‐world memory clinic, plasma p‐tau217 demonstrated high accuracy in discriminating patients with AD pathology (as determined by new Core1 biomarkers) from those with non‐AD pathology, with an AUC of 0.92. Our result is in line with previous recent works.^29^ Janelidze et al. described that plasma p‐tau217, when quantified using a mass spectrometry–based assay, achieved very high accuracy (AUC > 0.93) in identifying carriers of Aβ pathology and also those who progress to AD dementia.^31^ Palmqvist et al. recently described that %p‐tau217 (calculated as the ratio between p‐tau217 to non‐phosphorylated tau) yielded a higher AUC (0.97) in detecting Aβ pathology in secondary care.^12^ Moreover, plasma %p‐tau217 demonstrated excellent performance in identifying Aβ pathology (AUC 0.97) even among cognitively unimpaired individuals.^11^, ^32^ The slightly lower AUC found in our cohort might be explained by differences in methodology, as other studies used mass spectrometry or calculated %p‐tau217, which appears to have a better performance than what has been reported when using plasma p‐tau217 immunoassay.^6^, ^32^

To evaluate the real‐world applicability of plasma p‐tau217 as a peripheral marker of AD pathology, we tested two different approaches. First, we defined a single cut‐off of 0.274 pg/mL, which yielded an accuracy of 86.29%, a PPV of 88.18, and a NPV of 83.09%. The cut‐off identified in our cohort was similar to those reported in previous works.^16^, ^33^ Plasma p‐tau217 values were classified in positive and negative according to the cut‐off, showing good concordance with Core 1 status (i.e., presence or absence of AD pathology); however, a discordance was observed in 13% of cases, with 6% of false negatives and 7% of false positives. The use of a single cut‐off of plasma p‐tau217 seemed to be obviously suboptimal. The scenario drastically changed when the two cut‐offs approach was applied, improving the performance of plasma p‐tau217. In line with the Global CEO initiative on AD, which recommended to meet simultaneous goals of sensitivity and specificity > 90% and an intermediate results percentage of ≤ 20% for diagnostic tests on BBMs in secondary care,^23^ we examined three selected combinations of sensitivity and specificity: 95/95, 92/96, and 90/90.^24^ In our cohort, the 95/95 and the 92/96 combinations significantly increased accuracy and PPV of plasma p‐tau217. Notably, with the 92/96 combination, plasma p‐tau217 achieved an excellent accuracy of 91.11%, a PPV of 96.25%, and a good NPV of 83.63%. The proportion of intermediate values was 22%, consistent with the Global CEO recommendations.^24^ Our results are in line with previous works demonstrating the superiority of the two cut‐offs approach over a single cut‐off in identifying AD pathology.^12^, ^32^ To summarize, our results demonstrated that the diagnostic performance of plasma p‐tau217 improved when applying a two cut‐offs approach, achieving excellent accuracy with recommended sensitivity and specificity thresholds, even in real‐world settings. This supports the clinical utility of using two cut‐offs for plasma p‐tau217 immunoassays, as it reduces false positives and false negatives, thereby preventing patient misclassification, especially in light of the future global use of DMTs.^24^ Indeed, if optimal thresholds were validated, clearly negative and clearly positive patients could potentially avoid invasive or expensive confirmatory tests. On the other hand, patients with plasma p‐tau217 levels falling within the “gray zone” might be referred for additional confirmatory testing or, in the future, integration with other BBMs that could provide conclusive evidence to resolve diagnostic uncertainty.^32^

Interestingly, in our cohort a significant portion of patients falling within the gray zone were SCD, and this is a challenging finding to discuss. The two cut‐offs approach undoubtedly helps minimize misclassification, allowing ambiguous cases to be assigned to the gray zone. The frequent presence of SCD patients in this category may reflect ongoing biomarker dynamics in individuals developing AD. This hypothesis aligns with the findings of Janelidze et al., who demonstrated that cognitively unimpaired subjects with limited amyloid accumulation on amyloid PET (just below the positivity threshold on amyloid PET) exhibited elevated p‐tau217 levels.^11^ Consequently, it has been suggested that slow Aβ accumulation seen even in some asymptomatic subjects with negative amyloid PET may influence BBM concentrations.^11^ The application of the two cut‐offs approach proves especially valuable in real‐world cohorts including SCD patients, as it prevents misclassification and highlights patients in the gray zone, who warrant longitudinal follow‐up and additional investigations for appropriate risk stratification.

Our study has some limitations. First, renal function was considered only as a dichotomous variable (presence or absence of renal failure) rather than a continuous measure; moreover, only one patient in our cohort had impaired renal function. Future studies should collect more detailed data, such as creatinine levels, to better assess the impact of renal failure on plasma biomarker concentration.^34^ Second, we did not include healthy controls, and the relatively small sample size may limit the study's power and generalizability. Moreover, not all patients underwent both CSF biomarkers analysis and amyloid PET scans. Nevertheless, this study also has notable strengths. It focused not only on MCI but also on SCD, an even earlier stage of cognitive decline, which is a key target for early identification of individuals at risk of developing AD dementia. Furthermore, it is among the first to evaluate plasma p‐tau217 using the two cut‐offs approach in a real‐world setting. Finally, patients’ classification as carriers or non‐carriers of AD was based on the new Revised Criteria, including the updated definition of Core1 biomarkers.^2^

In conclusion, our work confirms that plasma p‐tau217, even when measured via immunoassay, is an excellent diagnostic biomarker for AD and it should be integrated in the decision‐making algorithm that considers the clinical context.^12^ The application of a two cut‐offs approach improved the performance of plasma p‐tau217, in line with the recommendations of the Global CEO Initiative on AD regarding the required performance of BBMs for diagnosing AD. Our findings strongly support the feasibility of plasma p‐tau217 in real‐world memory clinics, facilitating its transition from research setting to clinical practice. This advancement enables the detection of AD from the earliest stages of cognitive decline and the identification of patients at highest risk of developing AD dementia, which seems to be the ideal group in which to intervene with DMTs, changing the future perspective of AD.

## CONFLICT OF INTEREST STATEMENT

The authors declare that they have no competing interests. Author disclosures are available in the supporting information.

## ETHICS STATEMENT

Study procedures and data analysis were performed in accordance with the Declaration of Helsinki and with the ethical standards of the committee on human experimentation of our institute. The study was approved by the local institutional review board (reference 15691oss). All individuals involved in this research agreed to participate and agreed to have details and results of the research about them published.

## Supporting information



Supporting Information

Supporting Information

## Data Availability

All study data, including raw and analyzed data, and materials that support the findings of this study are available from the corresponding author (B.N.) upon reasonable request.

## References

[1] Ossenkoppele R , van der Kant R , Hansson O . Tau biomarkers in Alzheimer's disease: towards implementation in clinical practice and trials. Lancet Neurol. 2022;21(8):726‐734. doi:10.1016/S1474-4422(22)00168-5 35643092

[2] Jack CR , Andrews JS , Beach TG , et al. Revised criteria for diagnosis and staging of Alzheimer's disease: Alzheimer's Association Workgroup. Alzheimers Dement. 2024;20(8):5143‐5169. doi:10.1002/alz.13859 38934362 PMC11350039

[3] van Dyck CH , Swanson CJ , Aisen P , et al. Lecanemab in early Alzheimer's disease. N Engl J Med. 2023;388(1):9‐21. doi:10.1056/NEJMoa2212948 36449413

[4] Mattsson‐Carlgren N , Janelidze S , Bateman RJ , et al. Soluble P‐tau217 reflects amyloid and tau pathology and mediates the association of amyloid with tau. EMBO Mol Med. 2021;13(6):e14022. doi:10.15252/emmm.202114022 33949133 PMC8185545

[5] Yu L , Boyle PA , Janelidze S , et al. Plasma p‐tau181 and p‐tau217 in discriminating PART, AD and other key neuropathologies in older adults. Acta Neuropathol. 2023;146(1):1‐11. doi:10.1007/s00401-023-02570-4 37031430 PMC10261204

[6] Palmqvist S , Janelidze S , Quiroz YT , et al. Discriminative accuracy of plasma phospho‐tau217 for Alzheimer disease vs other neurodegenerative disorders. JAMA. 2020;324(8):772‐781. doi:10.1001/jama.2020.12134 32722745 PMC7388060

[7] Arslan B , Zetterberg H , Ashton NJ . Blood‐based biomarkers in Alzheimer's disease—moving towards a new era of diagnostics. Clin Chem Lab Med. 2024;62(6):1063‐1069. doi:10.1515/cclm-2023-1434 38253262

[8] Sato C , Barthélemy NR , Mawuenyega KG , et al. Tau kinetics in neurons and the human central nervous system. Neuron. 2018;97(6):1284‐1298. doi:10.1016/j.neuron.2018.02.015 29566794 PMC6137722

[9] Gonzalez‐Ortiz F , Ferreira PCL , González‐Escalante A , et al. A novel ultrasensitive assay for plasma p‐tau217: performance in individuals with subjective cognitive decline and early Alzheimer's disease. Alzheimers Dement. 2024;20(2):1239‐1249. doi:10.1002/alz.13525 37975513 PMC10916963

[10] Teunissen CE , Thijssen EH , Verberk IMW . Plasma p‐tau217: from “new kid” to most promising candidate for Alzheimer's disease blood test. Brain. 2020;143(11):3170‐3172. doi:10.1093/brain/awaa329 33278818 PMC7719020

[11] Janelidze S , Barthélemy NR , Salvadó G , et al. Plasma phosphorylated tau 217 and Aβ42/40 to predict early brain Aβ accumulation in people without cognitive impairment. JAMA Neurol. 2024;81(9):947‐957. doi:10.1001/jamaneurol.2024.2619 39068669 PMC11284634

[12] Palmqvist S , Tideman P , Mattsson‐Carlgren N , et al. Blood biomarkers to detect Alzheimer disease in primary care and secondary care. JAMA. 2024;332(15):1245‐1257. doi:10.1001/jama.2024.13855 39068545 PMC11284636

[13] Quaresima V , Pilotto A , Trasciatti C , et al. Plasma p‐tau181 and amyloid markers in Alzheimer's disease: a comparison between Lumipulse and SIMOA. Neurobiol Aging. 2024;143:30‐40. doi:10.1016/j.neurobiolaging.2024.08.007 39208716

[14] Mendes AJ , Ribaldi F , Lathuiliere A , et al. Head‐to‐head study of diagnostic accuracy of plasma and cerebrospinal fluid p‐tau217 versus p‐tau181 and p‐tau231 in a memory clinic cohort. J Neurol. 2024;271(4):2053‐2066. doi:10.1007/s00415-023-12148-5 38195896 PMC10972950

[15] Pilotto A , Quaresima V , Trasciatti C , et al. Plasma p‐tau217 in Alzheimer's disease: lumipulse and ALZpath SIMOA head‐to‐head comparison. medRxiv. 2024. doi:10.1101/2024.05.02.24306780 PMC11788209 39679606

[16] Cecchetti G , Agosta F , Rugarli G , et al. Diagnostic accuracy of automated Lumipulse plasma pTau‐217 in Alzheimer's disease: a real‐world study. J Neurol. 2024;271(10):6739‐6749. doi:10.1007/s00415-024-12631-7 39174818

[17] McKhann GM , Knopman DS , Chertkow H , et al. The diagnosis of dementia due to Alzheimer's disease: recommendations from the National Institute on Aging‐Alzheimer's Association workgroups on diagnostic guidelines for Alzheimer's disease. Alzheimers Dement. 2011;7(3):263‐269. doi:10.1016/j.jalz.2011.03.005 21514250 PMC3312024

[18] Albert MS , DeKosky ST , Dickson D , et al. The diagnosis of mild cognitive impairment due to Alzheimer's disease: recommendations from the National Institute on Aging‐Alzheimer's Association workgroups on diagnostic guidelines for Alzheimer's disease. Alzheimers Dement. 2011;7(3):270‐279. doi:10.1016/j.jalz.2011.03.008 21514249 PMC3312027

[19] Jessen F , Amariglio RE , van Boxtel M , et al. A conceptual framework for research on subjective cognitive decline in preclinical Alzheimer's disease. Alzheimers Dement. 2014;10(6):844‐852. doi:10.1016/j.jalz.2014.01.001 24798886 PMC4317324

[20] Mazzeo S , Lassi M , Padiglioni S , et al. PRedicting the EVolution of SubjectIvE Cognitive Decline to Alzheimer's Disease With machine learning: the PREVIEW study protocol. BMC Neurol. 2023;23(1). doi:10.1186/s12883-023-03347-8 PMC10422810 37573339

[21] Alcolea D , Pegueroles J , Muñoz L , et al. Agreement of amyloid PET and CSF biomarkers for Alzheimer's disease on Lumipulse—PubMed. Ann Clin Transl Neurol. 2019;6(9):1815‐1824. 31464088 10.1002/acn3.50873 PMC6764494

[22] Hansson O , Blennow K , Zetterberg H , Dage J . Blood biomarkers for Alzheimer's disease in clinical practice and trials. Nat Aging. 2023;3(5):506‐519. doi:10.1038/s43587-023-00403-3 37202517 PMC10979350

[23] Schindler SE , Galasko D , Pereira AC , et al. Acceptable performance of blood biomarker tests of amyloid pathology—recommendations from the Global CEO Initiative on Alzheimer's disease. Nat Rev Neurol. 2024;20(7):426‐439. doi:10.1038/s41582-024-00977-5 38866966

[24] Figdore DJ , Griswold M , Bornhorst JA , et al. Optimizing cutpoints for clinical interpretation of brain amyloid status using plasma p‐tau217 immunoassays. Alzheimers Dement. 2024;20(9):6506‐6516. doi:10.1002/alz.14140 39030981 PMC11497693

[25] Thijssen EH , La Joie R , Strom A , et al. Plasma phosphorylated tau 217 and phosphorylated tau 181 as biomarkers in Alzheimer's disease and frontotemporal lobar degeneration: a retrospective diagnostic performance study. Lancet Neurol. 2021;20(9):739‐752. doi:10.1016/S1474-4422(21)00214-3 34418401 PMC8711249

[26] Dyer AH , Dolphin H , O'Connor A , et al. Performance of plasma p‐tau217 for the detection of amyloid‐β positivity in a memory clinic cohort using an electrochemiluminescence immunoassay. Alzheimers Res Ther. 2024;16(1):186. doi:10.1186/s13195-024-01555-z 39160628 PMC11331802

[27] Therriault J , Janelidze S , Benedet AL , et al. Diagnosis of Alzheimer's disease using plasma biomarkers adjusted to clinical probability. Nat Aging. 2024;4(11):1529‐1537. doi:10.1038/s43587-024-00731-y 39533113 PMC11564087

[28] Ashton NJ , Puig‐Pijoan A , Milà‐Alomà M , et al. Plasma and CSF biomarkers in a memory clinic: head‐to‐head comparison of phosphorylated tau immunoassays. Alzheimers Dement. 2023;19(5):1913‐1924. doi:10.1002/alz.12841 36370462 PMC10762642

[29] Martínez‐Dubarbie F , Guerra‐Ruiz A , López‐García S , et al. Diagnostic accuracy of plasma p‐tau217 for detecting pathological cerebrospinal fluid changes in cognitively unimpaired subjects using the lumipulse platform. J Prev Alzheimers Dis. 2024;11(6):1581‐1591. doi:10.14283/jpad.2024.152 39559871 PMC11573816

[30] Therriault J , Vermeiren M , Servaes S , et al. Association of phosphorylated tau biomarkers with amyloid positron emission tomography vs tau positron emission tomography. JAMA Neurol. 2023;80(2):188‐199. doi:10.1001/jamaneurol.2022.4485 36508198 PMC9856704

[31] Janelidze S , Bali D , Ashton NJ , et al. Head‐to‐head comparison of 10 plasma phospho‐tau assays in prodromal Alzheimer's disease. Brain. 2023;146(4):1592‐1601. doi:10.1093/brain/awac333 36087307 PMC10115176

[32] Barthélemy NR , Salvadó G , Schindler SE , et al. Highly accurate blood test for Alzheimer's disease is similar or superior to clinical cerebrospinal fluid tests. Nat Med. 2024;30(4):1085‐1095. doi:10.1038/s41591-024-02869-z 38382645 PMC11031399

[33] Howe MD , Britton KJ , Joyce HE , et al. Clinical application of plasma P‐tau217 to assess eligibility for amyloid‐lowering immunotherapy in memory clinic patients with early Alzheimer's disease. Alzheimers Res Ther. 2024;16(1):154. doi:10.1186/s13195-024-01521-9 38971815 PMC11227160

[34] Janelidze S , Barthélemy NR , He Y , Bateman RJ , Hansson O . Mitigating the Associations of kidney dysfunction with blood biomarkers of Alzheimer disease by using phosphorylated tau to total tau ratios. JAMA Neurol. 2023;80(5):516‐522. doi:10.1001/jamaneurol.2023.0199 36987840 PMC10061310

