# Supplementary material for: The two cut‐offs approach for plasma p‐tau217 in detecting Alzheimer's disease in subjective cognitive decline and mild cognitive impairment
Source: Alzheimers Dement (Amst). 2025 May 11;17(2):e70116. doi: 10.1002/dad2.70116 (PMC12066393; doi:10.1002/dad2.70116)
Supplement: Supplementary file 1 — Supporting Information [file DAD2-17-e70116-s001.docx]

**Supplementary Materials 1**

**Collection of AD biomarker**

The CSF samples were collected by lumbar puncture, then immediately centrifuged and stored at −80 °C until performing the analysis. Aβ1–42, Aβ42/40 ratio, t-tau, and p-tau were measured using a chemiluminescent enzyme immunoassay (CLEIA) analyzer LUMIPULSE G600 (Lumipulse Beta Amyloid1–40, Lumipulse Beta Amyloid1–42, Lumipulse GTotal Tau, and Lumipulse GPhospho Tau (181)). Cut-offs for normal values were: for Aβ1–42, > 670 pg/mL; Aβ42/40 ratio, > 0.062; t-tau, < 400 pg/mL; and p-tau, < 60 pg/mL^1^. Reagent kits were obtained from Fujirebio.

Amyloid PET imaging was performed according to national and international standards ^2^, with any of the available fluorine18-labeled tracers (18Florbetaben [FBB]-Bayer-Pyramal, 18Flutemetamol [FMM]-General Electric). Images were rated as either positive or negative according to criteria defined by the manufacturers.

**Apolipoprotein E ε4 genotyping**

A standard automated method (QIAcube, QIAGEN) was used to isolate DNA from peripheral blood samples. *APOE* genotypes were investigated by high-resolution melting analysis (HRMA) ^3^. Two sets of PCR primers were designed to amplify the regions encompassing rs7412 [NC_000019.9:g[M13] [GG14] .45412079C>T] and rs429358 (NC_000019.9:g.45411941T>C). The samples with known *APOE* genotypes, which had been validated by DNA sequencing, were used as standard references. *APOE* genotype was coded as *APOE* ε4- (no *APOE* ε4 alleles) and *APOE* ε4+ (presence of one or two *APOE* ε4 alleles).

**Statistical Analysis**

Considering the first combination [95/95], the upper cutoff was set at a value yielding a specificity of 95%, while maximizing sensitivity, and the lower cutoff was set at a value resulting in a sensitivity of 95%, while maximizing specificity. Similarly, in the second combination [92/96], the upper cutoff was set at a value yielding a specificity of 96%, while maximizing sensitivity, and the lower cutoff was set at a value resulting in a sensitivity of 92%, while maximizing specificity. Finally, with the last combination [90/90], the upper cutoff was set at a value yielding a specificity of 90%, while maximizing sensitivity, and the lower cutoff was set at a value resulting in a sensitivity of 90%, while maximizing specificity. Participants with biomarker levels between these two thresholds were categorized as intermediate. For this approach, we compared the accuracy, sensitivity, specificity, PPV and NPV and the number of patients categorized as intermediate; accuracy, PPV and NPV only took into account patients in the negative and positive groups.

REFERENCES

1. Alcolea D, Pegueroles J, Muñoz L, et al. Agreement of amyloid PET and CSF biomarkers for Alzheimer’s disease on Lumipulse. *Annals of Clinical and Translational Neurology*. 2019;6(9):1815-1824. doi:10.1002/acn3.50873

2. Minoshima S, Drzezga AE, Barthel H, et al. SNMMI Procedure Standard/EANM Practice Guideline for Amyloid PET Imaging of the Brain 1.0. *J Nucl Med*. 2016;57(8):1316-1322. doi:10.2967/jnumed.116.174615

3. Sorbi S, Nacmias B, Forleo P, et al. ApoE allele frequencies in Italian sporadic and familial Alzheimer’s disease. *Neurosci Lett*. 1994;177(1-2):100-102. doi:10.1016/0304-3940(94)90054-x

**Supplementary Materials 2**

Demographic features and differences among diagnostic groups are summarized in Table 1. An AD-d patient and had impaired renal function (eGFR 44.6 mL/min/1.73 m^2^,), with no differences in terms of proportion of renal impairment among the SCD, MCI and AD-d groups.

Plasma p-tau217 concentration was correlated with age at plasma collection (Spearman’s ρ 0.219, *p*=0.003) and age at onset (Spearman’s ρ 0.291, *p*<0.001). Plasma p-tau217 levels were higher in AD-d as compared to MCI and to SCD patients (F [2,184]=23.35, *p*<0.001, partial η^2^=0.202), also after correcting for age (F [2,172]=20.71, *p*<0.001, partial η^2^=0.194) (Figure 1A). Plasma p-tau217 levels were slightly higher in *APOE* ɛ4 carriers than in non-carriers (0.80±0.67 vs 0.74±1.34, *p*=0.012). No differences in plasma p-tau217 levels were detected between males and females.

**Supplementary Table 1**

*Demographic features of Subjective Cognitive Decline (SCD), Mild Cognitive Impairment (MCI) and Alzheimer’s Disease dementia (AD-d) groups.*

|  | SCD | MCI | AD-d |
| --- | --- | --- | --- |
|  | N° 50 | N° 87 | N° 50 |
| Age at onset in years | **56.36 (±8.33) ^a,b^** | **65.36 (±9.83) ^a^** | **65.75 (±6.56) ^b^** |
| Age at plasma collection | **65.34 (±7.67) ^c,d^** | **69.96 (±8.54) ^c^** | **70.63 (±6.84) ^d^** |
| Family history of AD | 75.50% [63.46-87.54] | 60.50% [49.85-71.15] | 56.80% [42.16-71.44] |
| Sex (M – F) | 15 – 35 | 36 – 51 | 21 – 29 |
| Years of education | **13.41 (±3.64) ^e^** | **12.06 (±4.67)** | **10.83 (±4.73) ^e^** |
| MMSE | **28.69 (±1.40) ^f,g^** | **26.96 (±2.48) ^f,h^** | **19.56 (±4.90) ^g,h^** |
| *APOE* ɛ4+ | **23.40% ^i^ [11.30-35-50]** | 40.20% [29.59-50.81] | **53.30% ^i^ [38.72-67.88]** |
| Impaired renal function | 0 | 0 | 1 (2.00%) |
| Plasma p-tau217 (pg/ml) | **0.45 (±0.80) ^j^** | **0.49 (±0.52) ^k^** | **1.65 (±1.70) ^j,k^** |

Values are reported as mean and standard deviation or frequencies or percentages for continuous variables and categorical variables respectively. Statistically significantly different values between the groups are reported as underlined character. M: males; F: females; MMSE: Mini Mental State Examination. Statistically significance: *p*<0.05. ^a^ *p*<0.005; ^b^ *p*<0.001; ^c^ *p*=0.004; ^d^ *p*=0.003; ^e^ *p*=0.015; ^f^ *p*=0.006; ^g^ *p*<0.001; ^h^ *p*<0.001; ^i^ χ^2^ 8.73, *p*=0.005; ^j^ *p*<0.001; ^k^ *p*<0.001.

**Supplementary Table 2**

*Multiple regression analysis to predict plasma p-tau217 levels*

|  | B | 95% C.I. for B | | β | p |
| --- | --- | --- | --- | --- | --- |
|  |  | lower | upper |  |  |
| **Entire Cohort** |  |  |  |  |  |
| (Constant) | -0.346 | -0.813 | 0.121 |  | 0.145 |
| Diagnosis | 0.398 | 0.146 | 0.651 | 0.252 | 0.002 |
| Core1 status | 0.746 | 0.360 | 1.133 | 0.314 | <0.001 |
| **SCD and MCI** |  |  |  |  |  |
| (Costant) | 0.174 | 0.037 | 0.312 |  | 0.013 |
| Core1 status | 0.668 | 0.466 | 0.870 | 0.521 | <0.001 |

Unstandardized Regression Coefficients (B) and 95% Confidence Intervals (95% C.I.), standardized coefficient (β) and *p*-value (*p*), are reported (significant differences at *p*<0.05).
